# Adherence to insulin therapy and associated factors among type 1 and type 2 diabetic patients on follow up in Madda Walabu University Goba Referral Hospital, South East Ethiopia

**DOI:** 10.1371/journal.pone.0269919

**Published:** 2022-06-15

**Authors:** Feleke Hailu Chefik, Tesfaye Assefa Tadesse, Bruce John Edward Quisido, Adem Esmael Roba

**Affiliations:** Department of Nursing, School of Health Science, Goba Referral Hospital, Madda Walabu University, Bale Goba, Ethiopia; Università degli Studi di Milano, ITALY

## Abstract

**Background:**

Non-adherence to insulin therapy is a major global public health issue that has a causal relationship with increased diabetic complications that leads to further increase in the health care cost. However, adherence to insulin therapy and associated factors among diabetic mellitus (DM) patients are still not studied adequately in Ethiopia.

**Objective:**

To assess the adherence to insulin therapy and associated factors among type 1 and type 2 diabetic patients on follow-up at Madda Walabu University—Goba Referral Hospital, South East Ethiopia.

**Method:**

An institution-based, cross-sectional study was employed among 311 both type 1 and type 2 diabetic patients, Madda Walabu University—Goba Referral Hospital from March 4 to April 30, 2020. Study participants were recruited with simple random sampling method. Adherence to insulin therapy was measured by 8-item Morisky medication adherence scale. Therefore from these 8-items, those who score 6 or more are considered as adherent to insulin therapy. The data were collected through interviewer administered questionnaires by trained graduating class nurse students. The data were entered to Epidata version 3.1, and analyzed with SPSS version 25. Bivariate and multivariable logistic regression analyses were used to identify factors associated with adherence to insulin therapy. Statistical significance were declared at p <0.05.

**Result:**

A total of 311 patients participate in the study with response rate of 100%. Among these only 38.9% of them were adherent to insulin therapy with a CI of [33.5, 44.3]. Having glucometer (AOR = 3.88; 95% CI [1.46, 10.35]), regular hospital follow-up (AOR = 3.13; 95% CI [1.12, 8.70]), being knowledgeable (AOR = 3.36; 95% CI [1.53, 7.37]), and favorable attitudes (AOR = 4.55; 95%CI [1.68, 12.34]) were the factor associated with adherence to insulin therapy.

**Conclusion:**

This study concluded that adherence to insulin therapy was low in the study area. Having glucometer, regular hospital follow-up, being knowledgeable, and favorable attitudes were the factor associated with adherence to insulin therapy. Attention should be paid to help diabetic patients on acquiring knowledge regarding the need of consistent adherence to insulin therapy and its complications.

## Introduction

DM is recognized as the third leading cause of death in the world and become a significant public health issue [1, 2]. According to World Health Organization (WHO) estimation globally in 1980 and 2014–108 million and 422 million adults had DM with prevalence rate of 4.7% and 8.5%, respectively. Among these, 4 and 25 million with prevalence of 3.1% and 7.1% were in Africa [3]. Similarly, *i*n 2017, International Diabetes Federation (IDF) indicated 425 million adults were living with DM in 2015 with an estimated prevalence of 9.1% worldwide which will projected to shoot up to 693 million by 2045. Among this, 16 million were in the IDF region of Africa, which is expected to be 41.6 million by 2045. Ethiopia is the first among the 32 countries of IDF African region, where there are more than 2.5 million DM cases, with prevalence of 5.2% [4].

To date, along with this rapid increase in the number of diabetic cases, insulin has also prevailed as the most widely used treatment option for DM [5, 6]. On the downside, the treatment regimens for DM can be complex, which makes adherence to insulin therapy more difficult to be achieved in comparison to treatment regimens to other chronic diseases [7].

Adherence is defined as the extent to which a person’s behavior in taking medication, following a diet, and/or executing changes in lifestyle, in response toward the recommendations of health care providers. On the contrary, non-adherence is described as the extent to which a patient deviates from drug-taking behaviors which were recommended by health professionals [8]. As a result of non-adherence hospital admissions are increased by 30% contrary to those who are adherent [9].

Non-adherence to insulin therapy among patients with DM can be related to poor glycaemic control; that result in diabetic complications such as macro vascular (cardiovascular, peripheral, and cerebro-vascular diseases) and micro vascular (neuropathy, nephropathy, and retinopathy) [10, 11]. DM patients who are non-adherent are at high risk for all causes of hospitalizations (23.2%) and all causes of mortalities (5.9%) [12]. Insulin non-adherence has a causal relationship with diabetic complications that leads to further increase in the health care cost [13]. By 2015, the estimated cost of non-adherence to medications among DM patients ranged from 2,741 to 9,819 US dollars per individual patient [13].

To improve adherence level, understanding the extent of patient adherence and why non-adherence to insulin therapy occurs is important. Though studies conducted in various parts of the world regarding adherence to insulin therapy revealed 41.5% in Mexico City [14]. 43.1% in Iran [15], 62% [16] and 35.7% [17] in Saudi Arabia, 83.3% in Uganda [18], and 45.5% in Kenya [19]. In Ethiopia studies revealed that the level of adherence to insulin therapy were 61% at Jimma University Specialized Hospital [20], 66.93% at Tikur Anbessa Specialized Hospital [21], 59.2% at Felege Hiwot Referral Hospital [22], and 24.2% at some public hospitals in the central zone of Tigray [23].

Even though, adherence to insulin therapy is a crucial in the management of DM, the level of adherence to insulin therapy and factors associated with adherence to insulin therapy are not adequately throughout the country and the published articles are also confined to son part of the country. Therefore this study assess the level of adherence to insulin therapy and associated factors among type 1 and type 2 diabetic patients on follow up at MWU Goba referral hospital to bridge this information gap.

## Methods and materials

### Study design, setting and period

An institution based cross-sectional study design was conducted at Madda Walabu University—Goba Referral Hospital from March 4 to April 30, 2020 on DM patients that utilize insulin therapy.

### Source and study population

All type 1 and type 2 diabetic patients in Bale Zone receiving insulin therapy and have follow up at Madda Walabu University—Goba Referral Hospital were the source population, while all randomly selected type 1 and type 2 diabetic patients on insulin therapy and have follow up at the same hospital during the study period were considered as the study population.

### Inclusion and exclusion criteria

#### Inclusion criteria

All type 1 and type 2 diabetic patients on insulin therapy and have follow up for a period of at least six months were included in this study.

#### Exclusion criteria

The diabetic patients who were seriously ill and mentally unfit to participate in the interview, newly diagnosed, and those with age <15 years, were excluded.

#### Sample size and sampling procedure

Sample size was calculated by using single population proportion formula with assumption of the proportion of adherence to insulin therapy among diabetic patients from the study done previously in some public hospitals in the central zone of Tigray, Ethiopia, p = 24.2% [23] with 95% CI, 5% marginal error (d) was used. Then, considering 10% non-response rate, the final sample size became 311. According to the MWU GRH, District Health Information System-2 data, there were a total of 617 (384 Type I and 233 of Type II DM) patients who were getting insulin therapy. Simple random sampling approach was used to select samples from these groups of diabetic patients who are on insulin therapy.

### Study variables

The dependent Variable was ‘adherence to insulin therapy’ whereas; socio-demographic variable, health profile, knowledge and attitude regarding insulin therapy ware considered as Independent Variables.

### Data collection tools

The data collection tools were adapted from reviewed literatures with minimal modification, which was composed of five parts: Part I have nine items which aided to collect socio demographic data; Part II has eight-item Morisky Medication Adherence scale (8-MMAS) items used to measure the adherence levels; Part III is a twelve-item questionnaire which relates to the respondents’ health profiles; Part IV have eleven items that helped out to gather data related to knowledge of the study participants regarding insulin therapy; and finally, Part V, comprising of seven items which determined the attitude of the respondents toward insulin therapy.

Adherence to insulin therapy was measured by using the 8-MMAS which is a validated modified tool that contains 8 items regarding self-report measures that aid in assessing medication adherence on diabetes mellitus patients [24]. The first seven items on the 8-MMAS have response choices of "yes" or "no"–which were coded 0 for response “Yes” and 1 for response “No”. On the other hand, item number 5 was reversely coded to which the response “Yes” was coded as 1 and the response “No” was coded as 0. The last item had a 5-point scale of Likert scales, coded as 0 = Never, 1 = Awhile, 2 = Sometimes, 3 = usually and 4 = Always. In this case, the response 0 was rated as 1, while the rest of the responses 1, 2, 3 and 4 were rated as 0.

Data related to socio-demographic, health profile, knowledge and attitude about insulin therapy of the study participants were collected through structured questionnaire which were derived from the reviewed related literatures. The questionnaires were prepared in the English language and were translated to the local languages (Afan Oromo and Amharic) with the help of some native language speakers. Then after, the questionnaires were translated back to the English language for consistency.

### Data collection procedure and data quality assurance

The data were collected using face-to-face interviews conducted by three B.Sc. graduating class nursing students and were supervised by one B.Sc. nurse after a day of intensive training. To check for content suitability, clarity, sequence, and flow of the questions, the data collection tools were pre-tested on 5% of the sample size in a similar setting from the actual study area (Robe General Hospital), prior to the actual data collection. Necessary modifications were made based on the gaps identified in the data collection. Finally, the actual data were obtained from the study participants whom were selected thru simple random sampling method. At the end of each day of data collection, the questionnaires were reviewed and checked for completeness, accuracy and consistency by supervisor.

### Operational definition

#### Adherence to insulin therapy

Measured with the 8-MMAS. From these 8-MMAS, study participants who scored 6 or more were considered as adherent, while those who scored below 6 were considered as non-adherent to insulin therapy [14, 25].

#### Knowledge

Good knowledge is referred to as to the study participants who scored more than the mean value (≥6 or 54.5%) of the eleven knowledge-related questions, whereas those who scored below the mean value (≤5 or 45.5%) were considered as having poor knowledge.

#### Attitude

Favorable attitude is referred as to the study participants who scored above the mean value (≥4 or 57.1%) on the seven attitude-related questions, whereas those who scored below the mean value (≤3 or 42.9%) were considered as having unfavorable attitudes.

#### Data processing

The data were coded and entered to Epi Data version 3.1 and were analyzed thru SPSS version 25. Descriptive statistics was applied to present frequency distribution, of the study participants’ information. Multivariate logistic regression analysis was applied by selecting only those variables with P value ≤ 0.2 in the bivariate analysis to control confounding variables. Hosmer-Lemeshow test was used to check for accuracy of the logistic regression model. A confidence interval of 95% with p value of <0.05 was considered as statistically significant. The results of the study such as frequency distributions socio-demographic, health profile characteristics of study participants and bivariate and multivariate logistic regression analysis were presented in the form of table, while adherence level of the study participants is present in the form of figures.

### Ethics approval and consent to participants

Ethical clearance was obtained from the institutional review board of MWU-GRH. Since a high proportion of the study population had no formal education; verbal informed consent was obtained from each participant upon the data collectors were trained on how to implement the verbal consent and collected the data only if they consented for the interview. For participants less than 18 years of age assent was obtained from each individual and informed verbal consent was obtained from their families/guardians.

## Result

### Socio-demographic characteristics of the study participant

Overall 311 diabetic patients on insulin therapy were selected for this study with a response rate of 100%. Among these, 164 (52.7%) of the respondents were male and about 111 (35.7%) of them were found to be in the age category of 15–29 years old. Majority (66.2%) of the respondents were married and more than half (54%) of them were living in the rural areas. Additionally, 191 (61.4%) of the respondents were also followers of the Islam religion (Table 1).

**Table 1:** Frequency distributions of socio-demographic characteristics of study participants in Madda Walabu University Goba Referral Hospital Goba Referral Hospital (n = 311), 2020.

Variables	Category	Frequency (n)	Percent (%)
Age (in yrs.)	15–29	111	35.7
	30–45	106	34.1
	46–64	68	21.9
	>64	26	8.4
Sex	Male	164	52.7
	Female	147	47.3
Marital status	Unmarried	82	26.4
	Married	206	66.2
	Widowed	13	4.2
	Divorced	10	3.2
Residence	Urban	143	46
	Rural	168	54
Religion	Orthodox	106	34.1
	Islam	191	61.4
	Protestant	14	4.5
Ethnicity	Oromo	248	79.7
	Amhara	51	16.4
	Somali	6	1.9
	Others	6	1.9
Educational status	No formal education	107	34.4
	Grade 1–4	45	14.5
	Grade 5–8	54	17.4
	Grade 9–12	61	19.6
	≥diploma	44	14.1
Occupation	House wife	44	14.1
	Merchant	45	14.5
	Employee	37	11.9
	Farmer	137	44.1
	Students	37	11.9
	Other	11	3.5
Monthly income (Ethiopian Birr)	≤500	11	3.5
	501–1000	26	8.4
	1001–1500	36	11.6
	1501–2000	41	13.2
	≥2001	90	28.9
	Unknown	107	34.4

### Health profile of the study participants

From the patients that participated in this study, 106 (34.1%) of them had comorbidities–majority (63.2%) were hypertension. 202 (65%) of the respondents were identified to having type 1 DM. 132 (42.2%) of the respondents were <5 years from their diagnoses of being diabetics. In addition, (71.1%) of them were members of Ethiopian Diabetic Association and only a few had glucometers at home. Furthermore, 246 (79.1%) of the participants had regular hospital follow ups, but only 184 (59.2%) of them utilized insulin therapy (Table 2).

**Table 2:** Frequency distribution of health profile characteristics of study participants in Madda Walabu University Goba Referral Hospital (n = 311), 2020.

Variables	Responses	Frequency (n)	Percent (%)
Comorbidities	Yes	106	34.1
	No	205	65.9
If "Yes", which comorbidity/ies	Hypertension	67	63.2
	Heart failure	15	14.2
	Kidney disease	19	17.9
	Others	5	4.7
Type to DM	Type 1	202	65
	Type 2	109	35
Duration from diagnosis to DM	<5	132	42.4
	5 to 10	90	28.9
	10 to 15	42	13.5
	≥15	47	15.1
Membership to EDA	Yes	221	71.1
	No	90	28.9
Having a glucometer at home	Yes	49	15.8
	No	262	84.2
Checking blood glucose at home	Yes	40	81.6
	No	9	18.4
Duration of insulin therapy	<5	168	54
	5 to 10	93	29.9
	10 to 15	35	11.3
	≥15	15	4.8
Source of insulin	Purchasing	24	7.7
	DM association	198	63.7
	Insurance	85	27.3
	Others	4	1.3
Frequency of insulin self-injection	Once a day	14	4.5
	Twice a day	297	95.5
Regular follow up	Yes	246	79.1
	No	65	20.9
Taking alcoholic beverages	Yes	21	6.8
	No	290	93.2
Types of drugs	Only insulin	184	59.2
	Two types	67	21.5
	Three and above	60	19.3

### Level of adherence to insulin therapy

**Fig 1:**
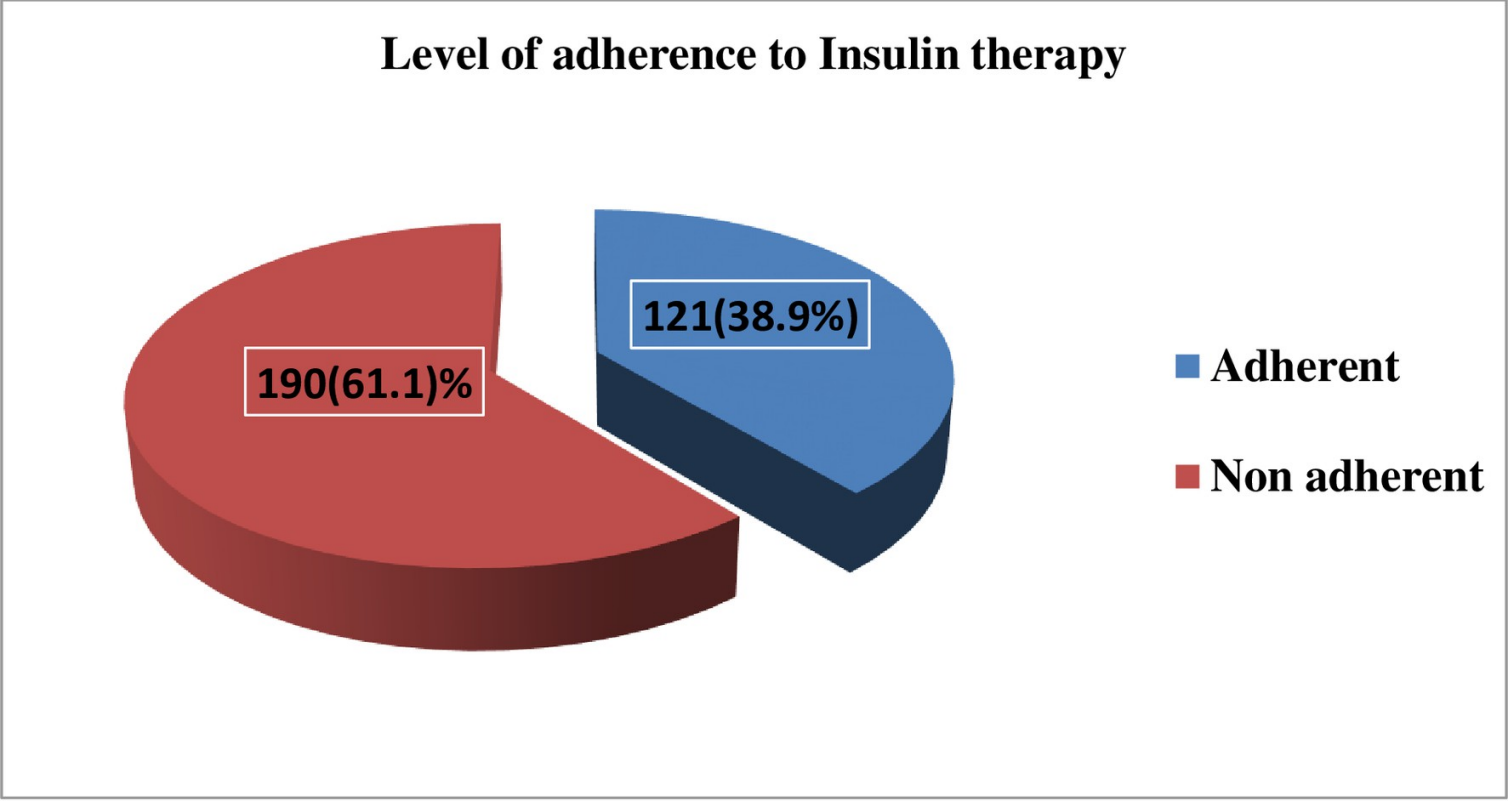
Adherence to insulin therapy level of the study participants in Madda Walabu University Goba Referral Hospital Goba Referral Hospital (n = 311), 2020.

Based on the scores from the 8-MMAS, the overall adherence levels to insulin therapy of the study participants were grouped to adherent and non-adherent. 121 (38.9%) with 95% CI [33.5, 44.3] of the respondents scored ≥6 and were considered as adherent, whereas the remaining with 95% CI of [55.7, 66.5] scored <6 and were considered as otherwise, non-adherent (Fig 1).

### Knowledge level and attitude of the study participants toward insulin therapy

Based on the scores from the eleven knowledge-related items regarding insulin therapy, 189 (60.8%) of the respondents scored ≥6 (54.5%) and were regarded as having good knowledge, while the rest (39.8%) who scored ≤5 (45.5%) were regarded as having poor knowledge.

In another context, based on the scores from the seven attitude-related items about insulin therapy, 219 (70.4%) of the respondents scored ≥4 (57.1%), enabling them to have favorable attitudes, while the remaining 92 (29.6%) who scored ≤3 (42.9%) were regarded as to having unfavorable attitudes.

### Factors associated with adherence to insulin therapy

Sixteen variables that had p values of ≤ 0.2 were identified using bivariate logistic regression. On multivariate logistic regression analysis, only seven variables remain as predictors of adherence to insulin therapy with 95% CI and significant levels of (p < 0.05).

Those respondents with type 2 DM were 77% times less likely to adhere to insulin therapy as compared to those with type 1 DM with (AOR = 0.23; 95%CI [0.07, 0.75]). Respondents who have glucometers were 3.88 times (AOR = 3.88; 95%CI [1.46, 10.35]) more likely to adhere to insulin therapy as compared to those of their counterparts. In addition, respondents who had regular hospital follow ups were 3.13 times (AOR = 3.13; 95%CI [1.12, 8.70]) more likely to adhere to insulin therapy than those who did not follow regularly. Furthermore, those who took two types of drugs were 69% times (AOR = 0.31; 95%CI [0.10, 0.96]) less likely to adhere to insulin therapy as equated to those who get sole insulin therapy.

Respondents who were diagnosed being diabetic for >15 years were 86% times (AOR = 0.14; 95%CI [0.04, 0.54]) less likely to adhere to insulin therapy as compared to those who were diagnosed for <5 years. The study participants that had good knowledge and favorable attitudes toward insulin therapy were 3.36 time (AOR = 3.36; 95%CI [1.53, 7.37]) and 4.55 times (AOR = 4.55; 95%CI [1.68, 12.34]) more likely to adhere to the therapy as compared to those who had poor knowledge and unfavorable attitudes (Table 3).

**Table 3:** Bivariate and multivariate logistic regression analyses of factors associated with adherence to insulin therapy among diabetic patients in MWU Goba Referral Hospital (n = 311), 2020.

Items	Response	Adherent		COR [95%CI]	AOR [95%CI]	P-value
		No	Yes			
Age category	15–29	48	63	1	1	
	30–45	69	37	0.41 [0 .24,.71]	0.71 [0.28,1.79]	0.474
	46–64	49	19	0.29 [0.15,.57]	2.60 [0.60,11.31]	0.202
	>64	24	2	0.06 [0.01,.28]	0.79 [0.08,7.99]	0.848
Residence	Urban	66	77	1	1	
	Rural	124	44	0.30 [0.19,.49]	1.10 [0.41,2.98]	0.845
Marital status	Unmarried	35	47	1	1	
	Married	134	72	0.40 [0.24, 0.68]	1.68[0.68,4.16]	0.259
	Divorced	8	2	0.19 [0.04, 0.93]	2.52 [0.32,20.16]	0.383
Religion	Orthodox	58	48	1	1	
	Islam	123	68	0.67 [0.41,1.08]	1.15 [0.53,2.48]	0.73
	Protestant	9	5	0.67 [0.21,2.14]	0.32 [0.07,1.49]	0.145
Educational status	No formal education	89	18	1	1	
	Grade 1–4	34	11	1.60 [0.69,3.73]	0.78 [0.26,2.39]	0.669
	Grade 5–8	31	23	3.67 [1.75,7.69]	0.78 [0.26,2.29]	0.65
	Grade 9–12	21	40	9.42 [4.53,19.58]	1.29 [0.41,4.03]	0.661
	≥diploma	15	29	9.56 [4.28,21.34]	1.09 [0.26,4.51]	0.91
Occupation	House wife	31	13	1	1	
	Merchant	26	19	1.74 [0.73,4.19]	0.34 [0.09,1.28]	0.111
	Employee	12	25	4.97 [1.93,12.78]	1.06 [0.23,4.90]	0.942
	Farmer	107	30	0.67 [0.31,.44]	0.36 [0.11,1.16]	0.086
	Students	9	28	7.42 [2.75,19.99]	3.36 [0.46,24.47]	0.514
	Other	5	6	2.86 [0.74,11.06	1.712 [0.34,8.67]	0.232
Comorbidities	Yes	81	25	1	1	
	No	109	96	2.85 [1.687,4.83]	0.53 [0.17,1.66]	0.272
Type of DM	Type 1	102	100	1	1	
	Type 2	88	21	0.24 [0.14, 0.42]	0.23 [0.07, 0.75]	0.015*
Member to EDA	Yes	128	93	1	1	
	No	62	28	0.62[0.37, 0.05]	0.78 [0.36,1.72]	0.547
Having glucometer	No	176	86	1	1	
	Yes	14	35	5.12 [2.62,10.01]	3.88 [1.46,10.35]	0.007*
Frequency of self-injection	Once a day	11	3	1	1	
	Twice a day	179	118	2.42 [.66,8.85]	1.51 [0.22,10.63]	0.678
Regular follow-up	No	56	9	1	1	
	Yes	134	112	5.20 [2.46,10.98]	3.13 [1.12,8.70]	0.029*
Number of drugs	Only insulin	90	94	1	1	
	Two types of drugs	52	15	0.28 [.15,.53]	0.31 [.10,.96]	0.043*
	≥3 drugs	48	12	0.24 [.12,.48]	0.64 [.13,3.18]	0.583
Duration from diagnosis to diabetes	<5	71	61	1	1	
	5 to 10	49	41	0.97 [.57, 1.67]	0.77 [.349,1.70]	0.519
	10 to 15	33	9	0.32 [.14,.72]	0.32 [.09,1.09]	0.068
	>15	37	10	0.32 [.15,.69]	0.14 [0.04,.54]	0.004*
Knowledge	Poor	102	20	1	1	
	Good	88	101	5.85 [3.35,10.23]	3.36 [1.53,7.37]	0.003*
Attitude	Unfavorable	84	8	1	1	
	Favorable	106	113	11.19 [5.17,24.20]	4.55 [1.68,12.34]	0.003*

* P value <0.05 for AOR, 1 = reference, AOR = Adjusted odds ratio, COR = Crude odds ratio.

## Discussion

This study revealed that the level of adherence to insulin therapy was 121 (38.9%) and were majorly influenced by the duration of diagnosis to diabetes of >15 yrs., unfavorable attitudes toward insulin therapy, being type 2 DM, not having glucometers at home, poor knowledge regarding insulin therapy, failing to follow regular hospital visits, and taking two types of drugs.

The level of adherence to insulin therapy with the current study had similarities from the findings done in some previous studies in Tertiary Care Center of Mexico City 41.5% [14], Tehran University of Iran 43.1% [15], and from a study in Bisha Primary Health Care Center of Saudi Arabia 35.7% [17], but had a slightly lower finding from a study done in Kenyatta National Hospital, Kenya 45.5% [19]. On another note, this finding was higher than the study conducted in some public hospitals of Central Zone, Tigray, Ethiopia 24.2% [23].

While the study findings were quite lower than those conducted in Asser Hospital, Saudi Arabia 62% [16], Bugiri Hospital of Eastern Uganda 83.3% [18], Felege Hiwot Referral Hospital, Bahir Dar 59.2% [22], Tikur Anbessa Specialized Hospital 66.93% [21], and Jimma University Specialized Hospital 61% [20], the discrepancies toward the adherence levels might have occurred due to the differences in times of the studies, sample sizes, health service utilization behaviors, life styles, and economic standards of the study participants.

This study indicated that those respondents who had favorable attitudes toward insulin therapy had increased their adherence to insulin therapy to 4.55 folds compared to those respondents who had unfavorable attitudes–this was supported by a finding from a previous study in some public hospitals of Central Zone of Tigray that favorable attitudes increase adherence levels to 2.14 times compared to those of unfavorable attitudes [23]. This might be due to the fact that having favorable attitudes help diabetic patients follow instructions from health workers better and increase their health seeking behaviors more.

According to the finding of the current study, having good knowledge regarding insulin therapy increase its adherence by multiples of 3.36 as compared to those respondents on their counterpart. This finding also has a similarity from the finding from a previous study conducted in some public hospitals in Central Zone of Tigray AOR of 4.21 [23]. This might be on account of the fact that having good knowledge can help the patients to take care of themselves, take their insulin properly, and take appropriate measures when they face undesirable conditions.

Those study participants who had glucometers at home increase their adherence to insulin therapy almost by four folds as equated to those who did not have them at home. This finding was accordant from a study done in some public hospitals of Central Zone of Tigray, that having glucometers at home augments adherence level to 2.81 times more as compared to its counterpart [23]. This might be because of the fact that patients who have glucometers at home are expected to check their blood sugars regularly, and for them to take actions to control their blood glucose levels. As a factor with a statistical significance, this study also identified that having regular hospital follow ups have significant associations toward adherence to insulin therapy.

Respondents who had regular follow ups increase their adherence to insulin therapy by factors of 3.13 as compared to those patients who did not have regular hospital visits. This was furthered by the report of ADA [25] and from a study done in Felege Hiwot Referral Hospital in Bahir Dar that those patients who have regular health care visits have 3.3 times more likelihood to adhere to insulin therapy as compared to its counterpart [22]. The reason behind might be because of regular follow ups create opportunities to have regular contacts with their health care providers that may help them to adjust their medication regimens with respect to their conditions, apart from getting professional counseling regarding their disease conditions and the treatments they are taking.

This study revealed that those respondents with type 2 DM decreased adherence to insulin therapy to 77% as compared to those respondents with type 1 DM. This finding was consistent from studies conducted in Tehran University of Iran [15] and Tikur Anbessa Specialized Hospital, Addis Ababa that type 2 DM patients were 25% times more likely to be non-adherent than those participants with Type 1 DM [26]. This might be owing to the reason that type 1 diabetic patients are younger in age, thus, minimizes their chances to forgetfulness; moreover, they easily understand the health education provided for them by health care providers, including any other means of information. In addition, those respondents with type 2 DM were subjected to multiple drug therapies which make their treatment regimens more complex.

This study also identified that taking two types of drugs are statistically significant factors that affect the adherence to insulin therapy and that they decrease the adherence by 69% in relation to those respondents that take only insulin. The result has a resemblance from the studies conducted at the Diabetic Clinic of Muhimbili National Hospital of Dar Salaam [2] and Debre Tabor General Hospital, Amhara Region, Ethiopia [27]. The reason might be due to decrease of complexities of the treatment regimens from taking insulin solely.

Diagnosis from Diabetes for >15 years was a statistically significant predictor of adherence to insulin therapy. It decreased the adherence to 86% compared to that of diagnosis for <5 years being diabetic–the study was congruent with the result from the study done in Kenyatta National Hospital, Kenya [19]. This could be by reason of boredom by those patients who had longer Diabetes Mellitus, as well as the treatment regimens that come along with them. Also, they may sometimes have tendencies for multiple drug therapies that make their treatment regimens more complex.

### Strength of the study

Madda Walabu University—Goba Referral Hospital established a chronic follow up clinic many years ago and has provided services for a large number of population in Bale Zone, enabling it to be considered as representative of the diabetic population in Bale. In addition, standardized tools were used to measure the adherence levels.

### Limitation of the study

Since this study was conducted using quantitative cross sectional study design, it cannot find possible cause-and-effect relationships between adherence levels and predictors.

## Conclusion

This study concluded that adherence to insulin therapy was low in the study area. Factors such as being type 2 DM, having glucometers at home, having regular hospital follow ups, taking two types of drugs, being diagnosed with DM for >15 years, being knowledgeable, and favorable attitude were also identified as the predictors of adherence to insulin therapy.

### Recommendations

A further large-scale study will be necessary to further the understanding of the issue and to design appropriate interventions. Since knowledge is a significant predictor of adherence to insulin therapy, MWU-GRH managers and the Bale Zone Health Office need to develop plans to provide diabetic health education to diabetic patients to develop their knowledge.

Health care workers should give ample emphasis in developing knowledge and attitude of diabetic patients regarding insulin therapy and its effects for non-adherence.

Ethiopian Diabetic Association in Bale Zone should promote and help diabetic patients to be diligent in attending their hospital appointments.

## Supporting information

S1 File (ZIP)

S2 File (ZIP)

## Abbreviations

CI: Confidence interval
DM: Diabetes Mellitus
GRH: Goba Referral Hospital
IDF: International Diabetic Federation
8-MMAS: 8 item Morisky Medication Adherence Scale
MWU: Madda Walabu University
WHO: World Health Organization
