# Supplementary material for: Adherence to insulin therapy and associated factors among type 1 and type 2 diabetic patients on follow up in Madda Walabu University Goba Referral Hospital, South East Ethiopia
Source: PLoS One. 2022 Jun 15;17(6):e0269919. doi: 10.1371/journal.pone.0269919 (PMC9200331; doi:10.1371/journal.pone.0269919)
Supplement: S2 File — (ZIP) [file pone.0269919.s002.zip › data collection tool.docx]

**Information sheet**

**Annex I: English version Questionnaires**

**Dear Respondent:**

My name is ____________________ I am working with Feleke Hailu who is currently adult health nursing postgraduate student in Madda Walabu University, Goba referral hospital, School of health science, Department of nursing. The aim of this study is to assess Adherence to insulin therapy and associated factor among patients with diabetes mellitus on follow up attending Madda Walabu University Goba referral hospital. The assessment is made for the partial fulfillment of Master’s Degree in adult health Nursing. The study will help to improve the adherence level of diabetic patients to insulin therapy based on your answers to the following questionnaire.

Therefore the data you will provide is very helpful to achieve the intended objectives of the study. The information obtained in this study will be used only for research purposes. Any information obtained will be kept strictly confidential and will not be exposed to any other body. Your name and any personal identifying term will not be written on the questionnaire. You will not face harm by participating and also not obliged to answer any question you don’t wish to answer. But your participation and contribution in the study is very important to come up with important findings which may help local health planners to intervene the problem.

Considering the information you get from the general information sheet, I would be Thankful if you spend some time to participate in the study. Do you agree to participate in this study?

If yes put right (☑) mark in the given box and continue to the next page and if no, say thank you and skip to the next participant

1. Yes 2. No

Name of the data collector ________________________Sign _________Date ___/___/______

**Data collection tools/questionnaire**

The data collection tool had 5 parts which was taken from various literatures with minimal manipulation to be fit with study.

- Part I- Part I have nine items which aided to collect socio demographic data; which was obtained from Ethiopian demographic health survey.
- Part II has 8-MMAS items used to measure the adherence levels; a validated tool that contains 8 items regarding self-report measures that aid in assessing medication adherence on diabetes mellitus patients. Taken from similar literature
- Part III is a twelve-item questionnaire which relates to the respondents’ health profiles; which was obtained from Ethiopian demographic health survey.
- Part IV have eleven items that helped out to gather data related to knowledge of the study participants regarding insulin therapy; which was obtained from similar literature
- Part V, comprising of seven items which determined the attitude of the respondents toward insulin therapy. Which was obtained from similar literature

**Part I Socio-demographic Characteristics of the respondents**

Please write the age and income of the respondent on the given space and for Q 102 – 109 encircle the respondent answers from the given alternatives

| **S.N** | **Questions** | **Response and Coding** | **Skip** |
| --- | --- | --- | --- |
| **101** | Age of respondent | ______________(in years) |  |
| **102** | Sex | 1. 1. Male 2. 2. Female |  |
| **103** | Residence | 1. 1. Urban area 2. 2. Rural area |  |
| **104** | Marital status | 1. Single  2. Married  3. widowed  4. Divorced  5. if other specify______________________ |  |
| **105** | Religion | 1. Orthodox  2. Muslim  3. protestant  4. if Others specify_____________________ |  |
| **106** | Ethnicity | 1. Oromo  2. Amhara  3. Somali  4. If Others specify____________________ |  |
| **107** | Educational status | 1. no formal education  2. Grade 1–4  3. Grade 5–8  4. Grade 9–12  5. College and above |  |
| **108** | Occupation | 1. House wife  2. Merchant  3. Employee  4. Farmer  6. If Others Specify_____________________ |  |
| **109** | Monthly income | __________________in Ethiopian birr |  |

**Part II. The 8 Item Morisky insulin therapy Adherence Scale.** For Q 201 – 107 encircle the respondent answers from the given alternatives and put (
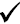
) under the given alternative of the respondents correct answer for Q-8

| S. N | Items | Response | | | | | | | Code | |
| --- | --- | --- | --- | --- | --- | --- | --- | --- | --- | --- |
| 201 | Do you sometimes forget to take your insulin? | 1) yes  2) no | | | | | | | 0 1 | |
| 202 | In the past two weeks, is there any days when you did not take all your prescribed insulin? | 1) yes  2) no | | | | | | | 0 1 | |
| 203 | Do you stop taking insulin when you feel worse? | 1) yes  2) no | | | | | | | 0 1 | |
| 204 | When you travel or leave home, do you sometimes forget to bring along your insulin? | 1) yes  2) no | | | | | | | 0 1 | |
| 205 | Did you take all your insulin as prescribed yesterday? | 1) yes  2) no | | | | | | | 1 0 | |
| 206 | When you feel like your diabetes is under control, do you sometimes stop taking your insulin? | 1) yes  2) no | | | | | | | 0 1 | |
| 207 | Did you ever feel hassled about sticking to your insulin regimen? | 1) yes  2) no | | | | | | | 0 1 | |
| 208 | How often do you have difficulty of remembering to take insulin? | 1  Never | | 2  Awhile | 3  Sometimes | | 4  Usually | | 5  Always | |
|  |  |  | |  |  | |  | |  | |
|  |  |  |  | | |  | |  | |  |

**Part III. Items related to health profile of the participants**

For Q 301 – 311 encircle the respondent answers from the given alternatives except (Q 304 & 305 put their response in a given space)

| S.No | Questionnaire | Response | Skip |
| --- | --- | --- | --- |
| 301 | Have you chronic disease other than DM? | 1. Yes 2. No | Q 303 |
| 302 | If yes for Q-301 which one? | 1. 1. Hypertension 2. 2. Heart failure 3. 3. Kidney disease 4. 4. Liver disease 5. 5. If other specify_________________ |  |
| 303 | Type of your Diabetes Mellitus | 1. Type 1 2. Type 2 |  |
| 304 | Duration of Diabetes diagnosis | ­­  ________________years |  |
| 305 | Duration on insulin therapy | _________________years |  |
| 306 | Are you member of Ethiopian diabetic association? | 1. Yes 2. No |  |
| 307 | Do you have glucometer at home? | 1. Yes 2. No | Q309 |
| 308 | If yes for Q-307 do you check your blood glucose? | 1. Yes 2. No |  |
| 309 | How do you get insulin? | 1. by purchasing  2. from diabetic association  3. by assurance  4. If other, specify_______________ |  |
| 310 | How often do you inject insulin your-self? | 1. Once a day  2. Twice per day  3. Three times per day  4. Four times per day  5. if other specify________________ |  |
| 311 | Have monthly regular follow up? | 1. Yes 2. No |  |
| 312 | Do you take alcohol drink? | 1. Yes 2. No |  |
| 313 | How many type of drug do you take? | 1. Only insulin 2. Two type 3 Three type 4. If others specify_____________ |  |

**Part IV: Knowledge Questionnaire regarding insulin therapy**

The following questions have only one possible correct answer please encircle the correct answer that the respondent choose

| S.N | Items | Response | Skip |
| --- | --- | --- | --- |
| 401 | Low blood glucose may be caused by: | 1. Too much insulin  2. Too little insulin  3. Too much food 4. I do not know |  |
| 402 | Which is inappropriate site for insulin vial is storage? | 1. refrigerator in the bottom  2. refrigerator in ice room  3. In sand soaked with water  4. I do not know |  |
| 403 | Massaging injection site after injection is used to Reduce the rapid absorption of insulin | 1. yes  2. no  3. I do not know |  |
| 404 | What is the purpose of rotating injection site? | 1. prevent lipohypertrophy  2. prevent hypoglycemia  3. prevent hyperglycemia  4. I do not know |  |
| 405 | Which one is not side effect of insulin? | 1. lipohypertrophy  2. hypoglycemia  3. hyperglycemia  4. I do not know |  |
| 406 | If you take your morning insulin but skip breakfast, Your blood glucose will be: | 1. Increase  2. Decrease  3. Remain the same  4. I do not know |  |
| 407 | High blood glucose may be caused by | 1. Not enough insulin dose  2. Skipping meals  3. Delaying your snack  4. I do not know |  |
| 408 | If you are beginning to have sign of insulin reaction, (hypoglycemia) what you will do? | 1. Lie down and rest  2. take sugar or candy  3. Take regular insulin  4. I do not know |  |
| 409 | Which one of the following will most likely cause an insulin reaction (hypoglycemia)? | 1. Heavy exercise  2. Overeating  3. Not enough insulin  4. I do not know |  |
| 410 | Insulin can be administered in the site of swelling or scar | 1. yes  2. no  3. I do not know |  |
| 411 | You realize just before lunch time that you forgot to take insulin before breakfast. What should you do? | 1. Skip lunch to lower your BGL 2. Take insulin that you usually take 3. Check your BGL to decide on how much insulin to take   4. I do not know |  |

**Part V. item related to attitude of participants towards adherence to insulin therapy**

Put (
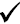
 ) under the given alternative for Q-501-507 based on the response of the respondents

| S.No | Items | 1 | 2 | 3 | 4 | 5 |
| --- | --- | --- | --- | --- | --- | --- |
|  |  | Strongly agree | Agree | Neutral | Disagree | Strongly disagree |
| 501 | Insulin self- injection is beneficiary |  |  |  |  |  |
| 502 | Insulin therapy is punishment of God due to our sin |  |  |  |  |  |
| 503 | Insulin self- injection is time consuming |  |  |  |  |  |
| 504 | Insulin self- injection bring stigma |  |  |  |  |  |
| 505 | Insulin cause other health problem |  |  |  |  |  |
| 506 | Once Insulin is started dietary management and exercise are not needed |  |  |  |  |  |
| 507 | Insulin can be stopped once blood glucose is controlled |  |  |  |  |  |

## Annexes II: Amharic version Questionnaires

**የመደ ወላቡ ዩኒቨርሲቲ ጎባ ሪፈራል ሆስፒታል**

**የጤና ሳይንስ ኮሌጅ**

**የነርሲንግ ትምህርት ክፍል**

የፈቃደኝነት መጠየቂያ ቅጽ

ጤና ይስጥልኝ

ስሜ --------------------------ይባላል በአሁኑ ሰዓት በመደ ወላቡ ዩኒቨርሲቲ፣ ጎባ ሪፈራል ሆስፒታል የድህረ ምረቃ ተማሪ ከሆነው ፈለቀ ኃይሉ ጋር አብሬ እሰራለሁ እግኛለሁ፡፡

የዚህ ጥናት ዋና አላማ በመደ ወላቡ ዩንቨርሲቲ፣ ጎባ ሪፈራል ሆስፒታል የስኳር ሕመም ክትትል የሚያደርጉ ቢኢንሱሊን የሚታከሙ ታካሚዎች ከመዳኒታቸው ጋር ያላቸውን ቁርኝት እና ከመዳኒት ቁርኝት ጋር ግንኙነተ ያላቸውን ጉዳዮች የሚዳስስ ይሆናል፡፡ ይህ ጥናት የሚከናወነው በአዋቂዎች ነርሲንግ ሙያ የሁለተኛ ዲግሪ ለመመረቅ ማሟያ ሢሆን፣ የጥናቱ ውጤት የስኳር ህመምተኞችን ጤና ለማሻሻል በእጅጉ ይጠቅማል፡፡ ስለሆነም ይህ መጠይቅ ጥናቱን ለማከናወን ጠቃሚ ስለሆነ እንዲሞሉ በትህትና ይጠየቃሉ፡፡ ይህን መጠይቅ ሲሞሉ ለሚስጥራዊነቱ ሲባል ስምዎም ሆነ ሌላ እርስዎን ሊገልጽ የሚችል ጉግይ በመጠይቁ ላይ አይጻፍም፡፡ የሚሰጡን መረጃ ሙሉ በሙሉ ሚስጠራዊነቱ ተጠብቆ ለጥናቱ ብቻ የሚውል ሲሆን በሂደቱ ላይ እንደአስፈላጊነቱ በሙሉ ነጻነት ይሙሉት ምንም ሊያስፈራዎት አይገባም ከዚህ በላይ ደግሞ እርስዎ መሙላት የማይፈልጉት ጥያቄ ቢኖር ለመሙላት አይገደዱም ይህን መጠይቅ ማቆም በሚፈልጉ ሰዓት ማቆም ይችላሉ ማቆሞት በእርሶ ህክምና ላይ ምንም ተጽእኖ አያመጣም፡፡

**የፈቃደኝነት ቅፅ**

ከላይ ባገኙት የመረጃ ምንጭ መሰረት ይህንን የምርምር ጥናት ጥያቄዎች ለመሳተፍ ፍቃደኝነትዎን እንጠይቃለን

በጥናቱ ለመሳተፍ ፍቃደኛ ነዎት

1. መልስዎ አዎ ከሆነ ወደ ሚቀጥለው ገጽ ይለፉ 2. መልስዎ አይደለሁም ከሆነ ወደ ሚቀጥለው ተሳታፊ ይሂዱ

መጠይቁን ያስሞላው፡ ስም ­­­­­­­­_____________ፊርማ _________ቀን___________

**SHEET CODE No ______ _____**

**ክፍል ፩፡ የተሳታፊው መረጃ**

ከ102-108 ላሉት ጥያቄዎች መልስ ሰጪው የመረጠውን ትክክለኛ መልስ ያክብቡ፤ ለጥያቄ 101 እና 109 በተሰጠው ባዶ ቦታ ላይ ትክክለኛውን መልስ ይጻፉ፡፡

| **ተ.ቁ** | **የአጠቃላይ ሁኔታ ጥያቄዎች** | **መልሶች** | **እለፍ** |
| --- | --- | --- | --- |
| **101** | እድሜዎ | ______________ዓመት |  |
| **102** | ጾታዎ | 1. ወንድ 2. ሴት |  |
| **103** | መኖሪያ ቦታ | 1. ገጠር 2. ከተማ |  |
| **104** | የጋብቻ ሁኔታ | 1. ያላገባ  2. ያገባ  3. የተፋታ (ች)  4. ሌላ ከሆነ ይግለጹ_______________ |  |
| **105** | ሃይማኖትዎ | 1. ኦርቶዶክስ  2. ሙስሊም  3. ፕሮቴስታንት  4. ሌላ ከሆነ ይግለጹ_______________ |  |
| **106** | ብሄርዎ | 1. ኦሮሞ  2. አማራ  3. ሶማሌ  4. ሌላ ከሆነ ይግለጹ________________ |  |
| **107** | የትምህርት ደረጃዎ | 1. መጻፍና ማንበብ የማልችል  2. ከ1 ኛ-4ኛ ክፍል  3. ከ 5ኛ-8ኛ ክፍል  4. ከ9ኛ- 12ኛ ክፍል  5. የኮሌጅ ዲፕሎማና ከዚያ በላይ |  |
| **108** | ስራ | 1. የቤት እመቤት  2. ገበሬ  3. ነጋዴ  4. የመንግስት ሰራተኛ  5. ሌላ ከሆነ ይግለጹ________________ |  |
| **109** | የወር ገቢዎ (በኢትዮጵያ ብር) | ___________________ ብር |  |

**ክፍል ፪. 8ቱ የሞሪስክይ የመዳኒት ቁርኝት መለኪያ**

ከ201-207 ላሉት ጥያቄዎች መልስ ሰጪው የመረጠውን ትክክለኛ መልስ ያክብቡ፤ ለጥያቄ 208 በተሰጡት አማራጮች ስር ባለው ሰንጠረዥ ላይ የ (
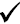
) ምልክት ያሰቀምጡ፡፡

| ተ.ቁ | መጠይቆች | | | መልሶች | | | ኮድ |
| --- | --- | --- | --- | --- | --- | --- | --- |
| 201 | አንዳንድ ጊዜ ኢነሱሊን መውሰድዎን ይረሳሉ? | | | 1) አዎ 2) የለም | | | 0 1 |
| 202 | ባለፉት ሁለት ሳምንታት ውስጥ የታዘዘውን የኢንሱሊን መጠን ያልወሰዱበት ቀን አለ? | | | 1) አዎ 2) የለም | | | 0 1 |
| 203 | ህመሙ የባስ ሲመስልዎት ኢነሱሊን መውሰድ ያቆማሉ? | | | 1) አዎ 2) የለም | | | 0 1 |
| 204 | አንዳንድ ጊዜ ጉዞ ላይ ሲሆኑ ኢነሱሊን መያዝ ይረሳሉ? | | | 1) አዎ 2) የለም | | | 0 1 |
| 205 | ትናንት በታዘዘው መሠረት ኢንሱሊን ወስደው ነበር? | | | 1) አዎ 2) የለም | | | 1 0 |
| 206 | ስኳርዎ የተስተካከለ ሲመስሎት ኢነሱሊን መውሰድ ያቆማሉ? | | | 1) አዎ 2) የለም | | | 0 1 |
| 207 | ህክምናዎትን ለመቀጠል ሲያስቡ የብስጭት ስሜት ይሰማዎተል? | | | 1) አዎ 2) የለም | | | 0 1 |
| 208 | ምን ያክል ጊዜ ኢንሱሊን ለመውሰድ የመርሳት ችግር ይገጥሞታል? | 1  በጭራሽ | 2  በጥቂቱ | | 3  አንዳንድ ጊዜ | 4  ብዙ ጊዜ | 5  ሁል ጊዜ |
|  |  |  |  | |  |  |  |

**ክፍል ፫፤ የተሳታፊዎች የጤና ሁኔታ መረጃ**

ከ301-313 ላሉት ጥያቄዎች መልስ ሰጪው የመረጠውን ትክክለኛ መልስ ያክብቡ፤ ለጥያቄ 303 እና 304 በተሰጠው ባዶ ቦታ ላይ ትክክለኛውን መልስ ይጻፉ፡፡

| ተ.ቁ | መጠይቅ | መልስ | እለፍ |
| --- | --- | --- | --- |
| 301 | ከስኳር ውጪ ሌላ ሥር የሰደደ ህመም አለብዎት? | 1) አዎ 2) የለም | ጥ303 |
| 302 | ለጥያቄ -303 መልስዎ አዎ ከሆነ የቱ ነው የለብዎት? | 1. የደም ግፊት  2. የልብ ድካም  3. የኩላሊት ህመም  4. ሌላ ከሆነ ይግለጹ_____________ |  |
| 303 | የትኛው ዓይነት የስኳር ህመም ነው ያለብዎት? | 1. አንደኛው 2. ሁለተኛው |  |
| 304 | የስኳር ህመም እንዳለብዎት ካወቁ ምን ያክል ሆነ? | _________ዓመት |  |
| 305 | ኢነሱሊን ከጀመሩ ምን ያክል ጊዜ ሆነ? | ___________ዓመት |  |
| 306 | የኢትዮጵያ ስኳር ህመምተኞች ማህብር አባል ነዎት? | 1) አዎ 2) የለም |  |
| 307 | የደም ስኳር መለኪያ አለዎት? | 1) አዎ 2) የለም | ጥ309 |
| 308 | ለጥያቄ-307 መልስዎ አዎ ከሆነ ስኳርዎትን ይለካሉ? | 1) አዎ 2) የለም |  |
| 309 | ኢነሱሊነ እነድት ነው የሚያገኙት? | 1. ከኢሹራንስ  2. ከስኳር ህመምተኞች ማህበር  3. በግዢ  4. ሌላ ከሆነ ይግለጹ__________ |  |
| 310 | በቀን ስንት ጊዜ ኢንሱሊን ይወጋሉ? | 1. በቀን 1 ጊዜ  2. በቀን 2 ጊዜ  3. በቀን 3 ጊዜ  4. ሌላ ከሆነ ይግለጹ __________ |  |
| 311 | በየወሩ ክትትል የህክምና ያገርጋሉ? | 1) አዎ 2) የለም |  |
| 312 | አልኮል መጠጥ ይጠጣሉ? | 1) አዎ 2) የለም |  |
| 313 | ስንት ዓይነት መድሀኒት ነው የሚወስዱት? | 1. ኢንሱሊን ብቻ  2. ሁለት ዓይነት  3. ሦስት ዓይነት  4. ሌላ ከሆነ ይግለጹ----- |  |

**ክፍል ፬፡** **የኢንሱሊንን በተመለከተ የተሳታፊዎች የእውቀት መለኪያ መጠይቆች** ለሚከተሉት ጥያቄዎች ከተሰጡት አማራጮች ተሳታፊው የሚሰጠውን መልስ ያክብቡ፡፡

| ተ.ቁ | ጥያቄዎች | መልስ | እለፍ |
| --- | --- | --- | --- |
| 401 | ከሚከተሉት ምክንያቶች የትኛው የደምን የስኳር መጠን ዝቅ ሊያደርግ ይችላል | 1. ከመጠን በላይ ኢንሱሊን መውሰድ 2. በጣም ትንሽ ኢንሱሊን መውሰድ 3. በጣም ብዙ ምግብ  4. አላውቅም |  |
| 402 | የኢንሱሊን ቤት ውሰጥ ለማስቀመት ተገቢ ያልሆነ ቦታው የትኛው ነው? | 1. ፍሪጅ ውስጥ ከታችኛው ክፍል  2. ፍሪጅ ውስጥ በበረዶ ክፍል  3. በውሃ የራሰ አሸዋ ውስጥ 4. አላውቅም |  |
| 403 | ኢንሱሊን ከተወጉ በኋላ የተወጋውን ቦታ ማሸት ኢንሱሊን በፍጥነት ወደ ደም ስር እንዳይገባ ያደርጋል | 1. አዎ 2. የለም 3. አላውቅም |  |
| 404 | ኢንሱሊን የሚወጉነትን ቦታ መቀያየር ለምን ይጠቅማል? | 1. የቆዳ ስር እብጠትን ለመከላከል 2. የደም ስኳር ማነስን ለመከላከል 3. የደም ስኳር መጨመርን ለመከላከል  4. አላውቅም |  |
| 405 | የኢንሱሊን የጎንዮሽ ጉዳት ያልሆነው የቱ ነው? | 1. የቆዳ ስር እብጠትን 2. የደም ስኳር መጠን ማነስ 3. የደም ስኳር መጠን መጨመር  4. አላውቅም |  |
| 406 | የጠዋት ኢንሱሊንዎን ወስደው ቁርስ ባይበሉ ፣ የደም የስኳር መጠን ምን ይሆናል? | 1. ይጨምራል 2. ይቀንሳል 3. ለውጥ አያመጣም  4. አላውቅም |  |
| 407 | ከሚከተሉት የትኛው የደም ስኳር መጠንን ሊጨምር ይጭላል | 1. በቂ ያልሆነ ኢንሱሊን መውሰድ 2. ምሳ አለመብላት 3. መክሰስ አለመብላት  4. አላውቅም |  |
| 408 | በኢንሱሊን ምክንያት የደም ስኳር መጠን ማነስ ስሜት ሲሰማዎት ምን ያደርጋሉ? | 1. እረፍት መውሰድ  2. ስኳር ወይም ከረሜላ ውሰድ  3. ውኃማውን ኢንሱሊን መውሰድ  4. አላውቅም |  |
| 409 | ከሚከተሉት ውስጥ የትኛው የደም ስኳር መጠን ማነስ ያመጣል? | 1. ከባድ የአካል ብቃት እንቅስቃሴ መስራት 2. ብዙ ምግብ መብላት 3. በቂ ያልሆነ ኢንሱሊን መውሰድ  4. አላውቅም |  |
| 410 | ኢንሱሊን ያበጠ ወይም ጠባሳ ያለበት ቦታ ላይ ሊሰጥ ይችላል | 1. አዎ 2. የለም 3. አላውቅም |  |
| 411 | ጠዋት የሚወስዱት ኢንሱሊን ቢረሱና መውሰድ ምሳ ሰዓት ላይ ቢያስታውሱ ምን ያደርጋሉ? | 1. የስኳር ለመቀነስ ምሳ አለመብላት 2. የጠዋቱን ኢንሱሊን መውሰድ 3. ምን ያህል የኢንሱሊን መውሰድ እንዳለበት ለመወሰን ስኳርን መለካት 4. አላውቅም |  |

**ክፍል ፭፡ የተሳታፊዎችን አመለካከት የሚዳስሱ መጠይቆች**

በተሳታፊዎቹ ምላሽን ላይ በመመርኮዝ ከ501-507 ላሉት ጥያቄዎች በተጠቀሰው አማራጭ ስር የ (
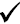
) ምልክት አስቀምጥ

| ተ.ቁ | ጥያቄዎች | 1 | 2 | 3 | 4 | 5 |
| --- | --- | --- | --- | --- | --- | --- |
|  |  | በጣም እስማማለሁ | እስማማለሁ | ገለልተኛ | አልስማመመም | በጣም አልስማመመም |
| 501 | ኢንሱሊን መወጋት ተጠቃሚ ነው |  |  |  |  |  |
| 502 | የኢንሱሊን ሕክምና በኃጢያታችን ምክንያት የእግዚአብሔር ቅጣት ነው |  |  |  |  |  |
| 503 | ኢንሱሊን መወጋት ብዙ ጊዜ ይፈጃል |  |  |  |  |  |
| 504 | ኢንሱሊን መወጋት ከህብረተሰቡ መገለልን ያመጣል |  |  |  |  |  |
| 505 | ኢንሱሊነ ሌላ የጤና ችግር ያመጣል |  |  |  |  |  |
| 506 | አንዴ ኢንሱሊን ከተጀመረ ምግብ ማስተካከልና የአካል ብቃት እነቅስቃሴ አያስፈልግም |  |  |  |  |  |
| 507 | የደም ስኳር መጠን ከተስተካከለ ኢንሱሊን ማቆም ይቻላል |  |  |  |  |  |

**ጥያቄዎቼን ጨርሻለሁ ስለ ትብብርዎ እና ጊዜዎትን መስዋዕት አድርገው በመጠይቆቹ ላይ ስለተሳተፉ በጣም አመሰግናለሁ!!!**

## Annexes III: Afan Oromo version Questionnaires

**YUUNIVARIISITII MADDA WALLAABUU,**

**HOOSPIITAALA RIIFARRAALLA GOOBAA**

**COOLEEJII FAYYAA**

**Ibsa wolii galtee**

Kabajamoo keenya fayyaaf nagaha keessanii?

Maqaan koo ____________________jedhama. Ani yeroo ammaatti barataa yuuniversiitii Madda Walaabuu muummee narsiingii digrii 2ffaa isaa adalt narsiingii dhaan (adult nursing) barachaa kan jiru Fallaqaa Hayiluu wajjiin hojjataa jiraachuu yoo ta'u, kaayyoon qorannoo kanaa haala wol fudhatiinsa qoricha insuuliinii jedhamuu fi wantoota isa wajjiin wal qabatan dhukkubsattota dukkuba sukkaaraa qaban kanneen yaala deddeebi'anii yaalamuu hospitaala riferaala Goobbaatti hordofan iratti madaallii gochuu ta'a.

Madaalliin kun kan adeemsifamuuf eebba barnoota digrii 2ffaa adalt narsiingii dhaan gaggeefamu kan ilaallatuu dha. Kan fayyaduu danda'us bu'uura fayyummaa hawaasaa deebii keessan gaaffii nuti isin gaaffannu irratti hundaa'uudhaan. Kanaafuu odeeffannoon isin nuuf kennitan galma ga'iinsa qorannoo kanaatiif baay'ee murteessaa kan ta'ee dha. Odeeffaannoon as keessaa argamu bu'aa qorannicha kanaa qofaaf kan oolu, iccitiidhaan kan eeggamuu, qaama biraatiif dabarfamee kan hin kennamne ykn hin laatamne, gama kanaan rakkoon tokkollee akka isin hin qunnamne, sodaa tokkollee akka hin qabaanne fi gaaffii deebii itti laachuu hin barbaannes akka irra dabruu dandeessan ifa isinii gochuu barbaada. Maqaa keessanis ta'ee waa'ee keessan kan ibsu odeeffannoon tokkollee akka hin baane dabalataan ibsuu barbaanna. Qorannoo kana keessatti hirmaachuu fi qooda fudhachuun keessan baay'ee barbaachisaa ta'uu fi bu'aa isaa immoo ogeeyyii naannoo waa'ee fayyaa irratti karoorsan akka rakkoo jiran furanii fi furmaata barbaadaniif gargaara.

Akka hubannoo odeeffanno fuula jalqabaa irraa argattanitti, baay'ee isin galateeffanna yoo yeroo muraasa nu wajjiin dabarsitan, gaaffii keenyaaf deebii laachuun dhimma jiru akka furmaata barbaannuuf yoo nu gargaartan.

Qorannoo kana keessatti hirmaachuuf hayyamamaadhaa?

1. Yoo eeyyen jettan, gama fuula itti aanuutti ce'aa
2. Yoo lakki jettan, bira dabraa gara hirmaataa itti aanuutti.

Maqaa nama daataa sassaabuu, _____________, mallattoo ____, guyyaa ________

**SHEET CODE No ______ _____**

**Kutaa 1ffaa. Akkaataa hawaassummaa fi fakkii ummataa hirmaataa.**

Gaaffilee armaan gadiitiif, maaloo deebii sirrii deebistoonni laatan qofa irra mari. akkasuumas gaafiilee 101fii109 deebii sirrii deebistoonni laatan bareesii

| T.L | Gaafilee | Deebii fi koodii | Yaada |
| --- | --- | --- | --- |
| 101 | Umrii | __________waggaadhaan |  |
| 102 | Saala | 1. 1. Dhiira 2. 2. Dhalaa |  |
| 103 | Iddoo jireenyaa | 1. 1. Magaalaa 2. 2. Baadiyaa |  |
| 104 | Haala fuudhaaf heerumaa | 1. 1. Kophaa 2. 2. Kan fuudhe/heerumte 3. 3. Kan jalaa du'e/jalaa duute 4. 4. Kan wal hiikan 5. 5. Kan biroo (ibsaa) __________ |  |
| 105 | Amantii | 1. 1. Oortoodoksii 2. 2. Musliima 3. 3. Prooteestaantii 4. 4. Kan biroo (ibsaa) _____ |  |
| 106 | Sabummaa | 1. 1. Oromoo 2. 2. Amaaraa 3. 3. Sumaalee 4. 4. KanBiroo (ibsaa) _______ |  |
| 107 | Sadarkaa barnootaa | 1. kan hiinbaranee  2. Baressuufii duubiisuu  3. kuuttaa1 -4 faa  4. kuuttaa5 -8 faa  4. kuuttaa 9-12 faa  5. koolejjii fii isaa olli |  |
| 108 | Gahee hojii | 1. 1. Haadha manaa 2. 2. Daldalaa/ttuu 3. 3. Hojjataa/ttuu mootummaa 4. 4. Qonnaan bulaa 5. 5. Kan biroo (ibsaa) _________ |  |
| 109 | Gallii ji’aa (qarshii Itoopiyaatiin) | qarshii ––––––––––––––––– |  |
|  | | | |

**Kutaa 2ffaa. Qabxiilee 8 kan wolfudhatiinsa yaala insuliinii xiinxallan ykn madaalan**

Gaaffilee armaan gadiitiif, maaloo deebii sirrii delebistoonni laatan qofa irra mari. Akkaasuuma gaaffii 208 deebii deebistootaa irratti hundaa'uun mallattoo (√) kaa'i

| T.L | Qabxiilee ykn gaafilee | | Deebii | | | | Code | |
| --- | --- | --- | --- | --- | --- | --- | --- | --- |
| 201 | Insuliinii fudhachuu yeroo tokko tokko ni dagattuu? | | 1) eeyyen  2) lakki | | | | 0 1 | |
| 202 | Torbaan dabran 2'n keessa insuliinii fudhattanii beektuu? | | 1) eeyyen  2) lakki | | | | 0 1 | |
| 203 | Yoo amalli hammaachuu sitti dhagahamu insuliinii addaan kuttee beettuu? | | 1) eeyyen  2) lakki | | | | 0 1 | |
| 204 | Yeroo tokko tokko yoo imala deemtu insuliinii dagattanii beettuu? | | 1) eeyyen  2) lakki | | | | 0 1 | |
| 205 | Kaleessa insuliinii fudhattanii? | | 1) eeyyen  2) lakki | | | | 1 0 | |
| 206 | Yoo sukkaarri sirritti siif too'atame insuliinii fudhachuu dhiiftanii beettuu? | | 1) eeyyen  2) lakki | | | | 0 1 | |
| 207 | Yaalli keessan itti fufaa ta'uu yoo yaaddan miirri aarii ykn uf hifannaa isinitti dhagahamaa? | | 1) eeyyen  2) lakki | | | | 0 1 | |
| 208 | Si'a meeqa akka insuliinii fudhattan yaadachuuf rakkattan? | 1= Tasumaa | | 2  Dabree dabree | 3  Yeroo muraasa | 4  Yeroo baay'ee | | 5  Yeroo hunda |
|  |  |  | |  |  |  | |  |

**Kutaa 3ffaa. Qabxiilee haala fayyummaa hirmaattotaa**

Gaaffilee armaan gadiitiif, maaloo deebii sirrii deebistoonni laatan qofa irra mari. akkasuumas gaafiilee 304-305 deebii sirrii deebistoonni laatan bareesii

| T.L | Gaafilee | Deebii | Yaada |
| --- | --- | --- | --- |
| 301 | Dhukkuba sukkaaraan ala dhibee biroo turaa qabduu? | 1) eeyyen 2) lakki | Yoo "lakki" ta'e gara gaaffii "303" dabraa |
| 302 | Yoo gaaffii "301"f eeyyen jettan, isa kam dhukkubsattu | 1. 1. Dhiibbaa dhiigaa 2. 2. Dhukkubbii onnee 3. 3. Dhukkubbii kalee 4. 4. Dhukkubbii tiruu 5. 5. Kan biroo (ibsaa) _____ |  |
| 303 | Sukkaarii kee isa kami? | 1. Isa 1ffaa dhaa  2. Isa 2ffaa dhaa |  |
| 304 | Dhukkubni sukkaara hanga beekkame turtii isaa | ____________waggaadhaan |  |
| 305 | Turtii hanga insuuliinii fudhachuu jalqabanii | ____________woggaadhaan |  |
| 306 | Isin miseensa waldaa dhukkuba sukkaara Itoophiyaa keessa jirtuu | 1) eeyyen 2) lakki |  |
| 307 | Maashinii safartuu dhukkuba sukkaaraa dhuunfatti manaa qabduu? | 1) eeyyen 2) lakki | Yoo gaaffii "309"f lakki ta'e gara 311tti cehaa |
| 308 | Yoo gaaffii "307"f eeyyen jettan, ufii keessan ilaaltanii beettuu? | 1) eeyyen 2) lakki |  |
| 309 | Insuliinii haala kamiin argattuu? | 1. 1. Ni bitadha 2. 2. Woldaa dhukkuba sukkaaraa 3. irraa fudha 3. 4. Inshuuraansii fayyaa irraa 4. 5. Kan biroo (ibsaa) ______ |  |
| 310 | Al ykn si'a meeqa waraannattu? | 1. 1. Guyyaan si'a tokko 2. 2. Guyyaan si'a lama 3. 3. Guyyaan si'a sadi 4. 4. Guyyaan si'a afur 5. Kan biroo (ibsaa) _________ |  |
| 311 | Dhukkuba sukkaaraa kanaaf ji'a ji'aan hordoffii dhaabbataa ni qabdaa ykn ni gootaa. | 1. 1) eeyyen 2. 2) lakki |  |
| 312 | Dhugattii alkoollii dhugduu? | 1. 1) eeyyen 2) lakki |  |
| 313 | Qooriicha goossa meeqaa fuudhatuu? | 1. Insuliinii qooffa  2. Goossa lamma  3. Goossa saddii  4 Kan biroo (ibsaa) _________ |  |

**Kutaa 4ffaa**. **Gaaffilee waa'ee beekumsa dhukkuba sukkaaraan wal qabatan**

Gaaffileen armaan gadii deebii sirrii tokkoo qofa qabu, maaloo deebii sirrii deebistoonni laatan qofa irra mari.

| T.L | Qabxiilee/ Gaaffilee | Deebii | darbii |
| --- | --- | --- | --- |
| 401 | Xiqqaachuun hamma sukkaara dhiiga keessaa kan mudachuu danda'uun sababa isa kamiin? | 1. Insuliiniin baay'achuudhaan  2. Insuliiniin hirdhachuudhaan  3. Nyaanni baay'achuudhaan  4. Hin beeku |  |
| 402 | Bilillee insuliinii kaayuuf iddoon sirrii hin taane isa kami? | 1. Qorrisiisaa gara jalaa  2. Qorrisiisaa kutaa cabbii  3. Bishaanitti cuubanii biyyee keessa kaa'uu  4. Hin beeku |  |
| 403 | Erga insuliinii waraannatanii iddoo san sukkuumuun insuliiniin akka dafee hin xuuxamne gargaara. | 1. Eeyyen  2. Lakki  3. Hin beeku |  |
| 404 | Faayidaan iddoo woraannatan jijjiirachuu maali? | 1. Akka faatiin hin furdanneef  2. Akka Sukkaarii hin hirdhanneef  3. Akka Sukkaarii hin baay'anneef  4. Hin beeku |  |
| 405 | Kamtu miidhaa insuliinii miti? | 1. Faatii furdisa  2. Hanqinna Sukkaara fida  3. Heddumminna Sukkaara fida  4. Hin beeku |  |
| 406 | Yoo insuliinii ganamaa fudhattee ciree kee dhiifte, hammi gulukoosii dhiiga keessaa maal ta'a? | 1. Ni dabala  2. Ni hirdhata  3. Akkasumatti tura  4. Hin beeku |  |
| 407 | Baay'achuun hamma sukkaara dhiiga keessaa kan mudachuu danda'uun sababa isa kami? | 1. Hammi insuliinii hirdhachuudhaan  2. Nyaata irra dabruudhaan  3. Nyaata tursiisuudhaan  4. Hin beeku |  |
| 408 | Yoo maallattoon hirdhachuu sukkaara dhiiga keessaa sirratti muldhachaa dhufe maal goota? | 1. Gadi jedhee boqonnaa fuddha  2. Sukkaara ykn karameellaaa fudha  3. Insuliinii bishaanii fudha  4. Hin beeku |  |
| 409 | Kanneen armaan gadii keessaa kamtu hirdhina sukkaara dhiiga keessaa fida? | 1. Sochii qaamaa ulfaataa dalaguu  2. Humnaa ol nyaachuu  3. Insuliinii gahaa hin taane fudhachuu  4. Hin beeku |  |
| 410 | Insuliiniin bakka iita'een ykn godaannisa qabuun ni keennama | 1. Eeyyen  2. Lakki  3. Hin beeku |  |
| 411 | Yeroo laaqanaatti insuliinii ciree duraa fudhachuu dhabuu kee yoo yaadatte maal goota? | 1. Sukkaara hirdhisuuf laaqana irra dabra  2. Insuliinima yeroo ciree fudhu san fudha  3. Hamma insuliinii fudhuu murteesssuuf dura hamma sukkaara dhiiga keessa beekuu qaba.  4. Hin beeku |  |

**Kutaa 5ffaa**. **Gaaffilee waa'ee ilaalcha dhukkuba sukkaaraatiin walqabatan**

Gaaffilee 501-507 deebii deebistootaa irratti hundaa'uun mallattoo (√) kaa'i

| T.L | Qabxiilee | 1 | 2 | 3 | 4 | 5 |
| --- | --- | --- | --- | --- | --- | --- |
|  |  | Sirritti fudha | Ni fudha | Homaa | Hin fudhu | Gonkumahin fudhu |
| 501 | Insuliinii ufii uf woraanuun bu'a qabeessa |  |  |  |  |  |
| 502 | Yaalli insuliinii sababa hojii keenyaatiif abaarsa waaqa irraa nutti dhufe |  |  |  |  |  |
| 503 | Insuliinii ufii uf woraanuun yeroo fudhata |  |  |  |  |  |
| 504 | Insuliinii ufii uf woraanuun kophatti nama baasa |  |  |  |  |  |
| 505 | Insuliiniin rakkoolee fayyaa kan biroo ni fida |  |  |  |  |  |
| 506 | Takka insuliinii jalqabnaan, nyaata sirreessuu ta'ee sochiin qaama baay'ee hin barbaachisu |  |  |  |  |  |
| 507 | Hammi sukkaara dhiiga keessaa yoo sirraa'e insuliiniin ni dhaabbata |  |  |  |  |  |

**Gaaffii koo xummureen jira. Yeroo keessan aarsaa gootanii turtii na wajjiin taasiftaniif guddaa galatoomaa. Fayyaa ta'aa, nagayatti!!!**
